# Predictive model for colorectal cancer stoma prolapse based on clinical characteristics: Construction path and validation system

**DOI:** 10.1097/MD.0000000000045090

**Published:** 2025-11-21

**Authors:** Fei An, Lin Gui, Yuting Li, Minjing Cheng

**Affiliations:** a Department of Gastrointestinal Surgery, The Third Hospital of Hebei Medical University, Shijiazhuang City, Hebei Province, China.

**Keywords:** colorectal cancer, intra-abdominal pressure, predictive model, stoma prolapse, type of stoma

## Abstract

In clinical practice, it is not possible to identify people at high risk of stoma prolapse during the perioperative and early postoperative periods and to implement preventive strategies based on the clinical characteristics of individual patients. This study investigates the risk factors for the development of stoma prolapse and to develop relevant predictive models. A total of 270 patients were collected in this study out of which 62 patients had stoma prolapse; the patients enrolled in this study were randomly divided into a training set and a validation set according to a ratio of 7:3, with 189 patients in the training set and 81 patients in the validation set. Information about the patients’ past medical history and hospitalization period was collected separately to study the correlates affecting the emergence of stoma prolapse in the patients and to establish a prediction model. Possibly relevant factors were included in a one-way logistic regression, and after analyzing the results: age, elevated intra-abdominal pressure, type of stoma, and hypoproteinemia were potential risk factors for the development of stoma prolapse during the 6-month postoperative period in patients who had undergone colorectal cancer stoma, *P* < .2; The data obtained were further included in a multifactorial review: age, elevated intra-abdominal pressure, type of stoma and hypo-proteinaemia were independent risk factors for stoma prolapse in patients with colorectal cancer stoma within 6 months after surgery, *P* < .05. This model provides clinicians with a powerful tool for early identification of patients at high risk of postoperative stoma prolapse. It helps to take targeted preventive and interventional measures before the onset of the disease.

## 1. Introduction

Globally, the incidence of colorectal cancer continues to climb and has become one of the major malignant tumors that seriously threaten human health.^[1–3]^ With the rapid development of diagnostic and therapeutic technologies, such as precise surgical protocols, highly effective radiotherapy and emerging targeted and immunotherapy, the survival period of colorectal cancer patients has been significantly extended. In this context, ostomy, as an important part of the treatment process of colorectal cancer, has been widely used to improve the intestinal excretory function of patients and provide essential survival support for some patients.^[4–8]^

However, the complication of postoperative stoma prolapse is a serious constraint to the quality of life and recovery process of patients. National and international studies have shown that the incidence of stoma prolapse in patients with colorectal cancer stoma ranges from 2% to 26%, a statistic that highlights the severity of the problem.^[9]^ In their study, Vasileios Papadopoulos’ team mentioned that the eventual incidence of stoma prolapse, a common late complication, depends to some extent on the systematic follow-up of the patient and the initial surgical technique.^[9]^ Stoma prolapse not only triggers physical discomfort in patients, such as local pain, bleeding, intestinal obstruction, etc, but also deals a heavy blow to the psychological state of patients, leading to the growth of anxiety, depression and other undesirable emotions, which greatly depletes healthcare resources and the social support system.^[9–11]^ At present, domestic and international research for colorectal cancer stoma prolapse has made some progress. In terms of exploring the pathogenesis, it is generally accepted that aging, chronically elevated intra-abdominal pressure, type of stoma (e.g., higher risk of prolapse in permanent stomas compared to temporary stomas), and hypoproteinemia are strongly associated.^[12,13]^ However, there are still many controversies and uncharted territories in the academic community regarding the pathophysiological mechanisms specific to colorectal cancer. For example, the influence of the tumor microenvironment on local tissue repair and support structures of the stoma, and whether colorectal cancer-related genetic polymorphisms increase the risk of stoma prolapse by influencing connective tissue metabolism and thereby increasing the risk of stoma prolapse, have not yet been fully elucidated.^[14,15]^ Predictive modeling plays a vital role in healthcare, not only by helping to prevent patient complications – such as stoma prolapse in colorectal cancer patients – but also by enabling more accurate forecasting of hospital resource needs. Early identification of high-risk individuals allows healthcare facilities to proactively manage staffing, equipment availability, and bed occupancy. This creates a crucial connection between personalized risk assessment and broader system-level planning, ultimately enhancing both patient care and operational efficiency.^[16]^ In clinical diagnosis and treatment, the existing prediction tools are mostly based on limited clinical indicators, which lack systematic and comprehensive, making it difficult to accurately identify patients at high risk of stoma prolapse. Although some studies have attempted to construct predictive models, the sensitivity and specificity of the models are poor, and their generalisability to different healthcare settings and patient populations is questionable. Recent studies abroad have attempted to combine machine learning algorithms and integrate multidimensional data to construct predictive models, but the generalization of the models has been hampered by the geographical limitations of the data samples and the heterogeneity of the diseases. In view of this, this study aims to provide an in-depth analysis of the clinical characteristics of colorectal cancer patients through clinical data collection. Construction and rigorous validation of a stoma prolapse prediction model using advanced statistical methods and data mining techniques. In order to provide strong support for early precision prevention and intervention, fill the research gaps in this field, and promote the standardization and precision of colorectal cancer stoma patient management.

## 2. Materials and methods

### 2.1. Research object

The subjects of this study were patients who consulted the Department of Gastrointestinal Surgery of the Third Hospital of Hebei Medical University from January 2021 to June 2024, were diagnosed with colorectal cancer and underwent colorectal cancer stoma in our department. The total number of study participants was 270, of whom 144 were men and 126 were women. Patients were informed about this study and signed the relevant informed consent forms with them. The study was approved by the Ethics Committee of our hospital. Number: W-2025-034-1.

### 2.2. Research methods

This study was a retrospective case-control study. Sixty-two of these patients developed stoma prolapse within 6 months of surgery. Information about the patients’ past medical history and hospitalization period was collected separately to study the correlating factors affecting the development of stoma prolapse in the patients within 6 months postoperatively.

### 2.3. Collection of indicators

Sex, age, BMI, history of smoking, history of alcohol consumption, hypertension, diabetes mellitus, coronary artery disease, increased intra-abdominal pressure, type of stoma, location of stoma, and occurrence of hypoproteinemia.

### 2.4. Inclusion and exclusion criteria

#### 2.4.1. Inclusion criteria

(1) Pathologically diagnosed patients who met the criteria of Chinese Colorectal Cancer Diagnostic and Treatment Guidelines (Version 2023) and who had a colostomy;
(2) The duration of the colostomy was more than 1 month;
(3) The age was ≥ 18 years old, and those who were aware of the disease and possessed the ability of normal verbal communication;
(4) The patients volunteered to participate and signed an informed consent form;
(5) The clinical data were complete and without any defects.

#### 2.4.2. Exclusion criteria

(1) Patients with other malignant tumors;
(2) Those with other serious complications of the heart, brain, kidney, lungs, etc;
(3) Those with previous psychiatric disorders or severe psycho-cognitive dysfunction.
(4) Patients who are noncompliant or refuse to participate in the study.

### 2.5. Interpretation of some indicators mentioned in this study

(1) **Measurement of intra-abdominal pressure:** The patient is placed in a supine position, and after emptying the bladder, a Foley catheter is inserted through the urethra. 25 to 50 mL of sterile saline solution is injected into the bladder, and a pressure sensor or pressure gauge is connected. Using the symphysis pubis as the zero point, the pressure inside the bladder is measured, and the measured value indirectly reflects the intra-abdominal pressure.
(2) **Measurement of blood albumin:** Principle: Peptide bonds in proteins react with copper ions in alkaline solutions to form purple-red complexes, with the intensity of the color being directly proportional to the protein content. Procedure: Collect venous blood, separate the serum, and add biuret reagent (containing alkaline copper solution). Measure the absorbance at a wavelength of 540 to 560 nm using a spectrophotometer, and calculate the albumin concentration by comparing it with the standard curve.
(3) **Increased intra-abdominal pressure:** Increased intra-abdominal pressure is associated with a number of factors, such as constipation, chronic coughing due to diseases such as chronic obstructive pulmonary disease, and poor urination due to prostate enlargement.
(4) **Types of stoma:** An enterostomy is a surgical procedure in which a portion of the intestines is guided out of the abdominal wall to form an opening for excretion or decompression, and usually consists of a single-lumen stoma and a double-lumen stoma.
(5) **Stoma location:** The abdominal wall location of the enterostomy needs to be selected in conjunction with the location of the intestinal lesion, the purpose of the operation and other factors, including transrectus abdominis stoma and para-rectus abdominis stoma.
(6) **Definition of stoma prolapse:** Stoma prolapse is a long-term complication of intestinal stoma surgery, referring to the proximal intestinal tract protruding more than 5 centimeters through the stoma, with the intestinal loop protruding excessively from the abdominal skin, reaching lengths of several centimeters to tens of centimeters. Essentially, this is a case of the intestinal tract everting and prolapsing from the stoma, which may be accompanied by severe symptoms such as stoma edema, bleeding, ulceration, intestinal torsion, obstruction, and even ischemic necrosis.
(7) Diagnosis of stoma prolapse:
  ① Clinical manifestations: Protrusion of the intestinal tract at the stoma site ≥ 5 cm, worsening when standing or with increased abdominal pressure, and partially/completely reducible (temporary) or non-reducible (persistent) when lying down; accompanied by mucosal edema and bleeding, with severe cases presenting intestinal obstruction, torsion, or ischemic necrosis.
  ② Etiological factors: Surgical factors (excessively large stoma, improper positioning) or increased abdominal pressure triggers (coughing, constipation, obesity).
  ③ Physical examination findings: Visual inspection reveals intestinal prolapse; palpation assesses intestinal activity (color, tension, and impaction status).
  ④ Additional tests: Imaging studies (CT/ultrasound) to rule out stoma hernias or obstruction; laboratory tests to assess infection or electrolyte imbalances.

### 2.6. Statistical methods

The data was processed and statistically analyzed in this study using SPSS 25.0 (Chicago). Quantitative information that conformed to normal distribution was expressed as mean ± standard deviation, and differences between groups were analyzed using the independent samples *t*-test. Comparisons between groups that do not obey a normal distribution are made using nonparametric tests. Data for qualitative information were expressed as number of cases and percentages, and the chi-square test was used to determine if there were differences between groups. The patients were first randomly divided into a training set and a validation set in a 7:3 ratio. The incidence of stoma prolapse was then analyzed based on factors such as the occurrence of stoma prolapse in the training set of patients and various clinically relevant indicators. Potential risk factors for the development of stoma prolapse were subsequently identified based on a one-way logistic regression analysis of the collected data. For the univariate analysis, exposure factors with *P* < .05 were selected and included in the multivariate analysis. An independent risk factor for the development of stoma prolapse was derived, and a difference of *P* < .05 was considered statistically significant. The model was then internally validated to further confirm the reliability of the predictive model derived from this study.

## 3. Results

### 3.1. Table of values assigned to the relevant indicators in this study

In this study, the variables were assigned values based on the categorization of variables such as gender, stoma prolapse, increased intra-abdominal pressure, type of stoma, and location of stoma. For example, if the gender is female, the value 0 is assigned; if the gender is male, the value 1 is assigned. See Table 1 for details.

**Table 1:** Assignment table.

Name	Variable assignment and description
Gender	Female-0, male-1
Stoma prolapse	No-0, yes-1
Increased intra-abdominal pressure	No-0, yes-1
Type of stoma	Double-lumen stoma-0, Single-chamber stoma-1
Stoma position	Transrectus abdominis muscle stoma-0, Para-rectus abdominis stoma-1

### 3.2. Table of baseline characteristics

The general information of the patients in both groups was included in the statistical study, *P* > .05, and the differences in baseline characteristics between the 2 groups were not statistically significant. See Table 2 for details.

**Table 2:** Comparison of baseline features between training and validation sets.

Variables	Total (n = 270)	test (n = 81)	train (n = 189)	Statistic	*P*
BMI	23.15 ± 2.81	22.96 ± 2.55	23.22 ± 2.92	t = −0.69	.488
Stoma prolapse, n(%)					
0	208 (77.04)	63 (77.78)	145 (76.72)	χ²=0.04	.850
1	62 (22.96)	18 (22.22)	44 (23.28)		
Gender, n(%)					
0	126 (46.67)	35 (43.21)	91 (48.15)	χ²=0.56	.456
1	144 (53.33)	46 (56.79)	98 (51.85)		
Age, n(%)					
0	94 (34.81)	26 (32.10)	68 (35.98)	χ²=0.38	.540
1	176 (65.19)	55 (67.90)	121 (64.02)		
History of smoking, n(%)					
0	203 (75.19)	58 (71.60)	145 (76.72)	χ²=0.80	.373
1	67 (24.81)	23 (28.40)	44 (23.28)		
Drinking history, n(%)					
0	149 (55.19)	46 (56.79)	103 (54.50)	χ²=0.12	.728
1	121 (44.81)	35 (43.21)	86 (45.50)		
Hypertensive, n(%)					
0	186 (68.89)	58 (71.60)	128 (67.72)	χ²=0.40	.528
1	84 (31.11)	23 (28.40)	61 (32.28)		
Diabetes mellitus, n(%)					
0	232 (85.93)	68 (83.95)	164 (86.77)	χ²=0.37	.541
1	38 (14.07)	13 (16.05)	25 (13.23)		
Coronary heart disease, n(%)					
0	241 (89.26)	72 (88.89)	169 (89.42)	χ²=0.02	.898
1	29 (10.74)	9 (11.11)	20 (10.58)		
Increased intra-abdominal pressure, n(%)					
0	128 (47.41)	34 (41.98)	94 (49.74)	χ²=1.37	.242
1	142 (52.59)	47 (58.02)	95 (50.26)		
Stoma position, n(%)					
0	90 (33.33)	29 (35.80)	61 (32.28)	χ²=0.32	.573
1	180 (66.67)	52 (64.20)	128 (67.72)		
Type of stoma, n(%)					
0	126 (46.67)	38 (46.91)	88 (46.56)	χ²=0.00	.958
1	144 (53.33)	43 (53.09)	101 (53.44)		
Hypoproteinemia, n(%)					
0	125 (46.30)	32 (39.51)	93 (49.21)	χ²=2.15	.143
1	145 (53.70)	49 (60.49)	96 (50.79)		

BMI = body mass index.

### 3.3. One-way analysis of variance

The training set of 189 patients was included in the statistical analysis, of which 44 patients developed stoma prolapse. Possibly relevant factors were included in a one-way logistic regression and analyzed to give the result that age, elevated intra-abdominal pressure, type of stoma and hypo-proteinaemia are potential risk factors for stoma prolapse in patients with colorectal cancer stoma in the 6-month postoperative timeframe, *P* < .05. See Table 3 for details.

**Table 3:** One-factor logistic regression analysis based on training set.

Variables	β	S.E	Z	*P*	OR (95% CI)
Gender					
0	–	–	–	–	1.00 (Reference)
1	0.14	0.35	0.41	0.683	1.15 (0.59–2.27)
Age					
0	–	–	–	–	1.00 (Reference)
1	1.79	0.50	3.55	0.002	5.99 (2.23–16.08)
Smoking history					
0	–	–	–	–	1.00 (Reference)
1	0.28	0.39	0.71	0.475	1.32 (0.61–2.86)
Drinking history					
0	–	–	–	–	1.00 (Reference)
1	−0.12	0.35	−0.35	0.724	0.88 (0.45–1.75)
Hypertensive					
0	–	–	–	–	1.00 (Reference)
1	0.24	0.36	0.66	0.508	1.27 (0.63–2.58)
Diabetes mellitus					
0	–	–	–	–	1.00 (Reference)
1	−0.53	0.58	−0.92	0.360	0.59 (0.19–1.82)
Coronary heart disease					
0	–	–	–	–	1.00 (Reference)
1	0.39	0.52	0.75	0.454	1.48 (0.53–4.11)
Increased intra-abdominal pressure					
0	–	–	–	–	1.00 (Reference)
1	1.71	0.41	4.16	<.001	5.51 (2.47–12.30)
Stoma position					
0	–	–	–	–	1.00 (Reference)
1	-0.11	0.36	-0.29	0.769	0.90 (0.44–1.84)
Type of stoma					
0	–	–	–	–	1.00 (Reference)
1	1.71	0.42	4.03	<.001	5.54 (2.41–12.74)
Hypoproteinemia					
0	–	–	–	–	1.00 (Reference)
1	2.25	0.47	4.78	<.001	9.50 (3.78–23.91)
BMI	−0.03	0.06	−0.46	0.647	0.97 (0.87–1.09)

BMI = body mass index, CI = confidence interval, OR = odds ratio.

### 3.4. Multifactorial analysis

The 4 risk factors derived from the univariate analysis of this study were further included in the multivariate analysis, which showed that age, elevated intra-abdominal pressure, type of stoma and hypo-proteinaemia were independent risk factors for stoma prolapse in patients with colorectal cancer stoma during the 6 months postoperative time. See Table 4 for details.

**Table 4:** Multifactor logistic regression analysis based on training set.

Variables	β	S.E	Z	*P*	OR (95% CI)
Intercept	−5.00	0.77	−6.49	<.001	0.01 (0.00–0.03)
Age					
0	–	–	–	–	1.00 (Reference)
1	1.69	0.55	3.07	0.002	5.39 (1.84–15.83)
Increased intra-abdominal pressure					
0	–	–	–	–	1.00 (Reference)
1	1.26	0.46	2.74	0.006	3.51 (1.43–8.63)
Type of stoma					
0	–	–	–	–	1.00 (Reference)
1	1.01	0.51	1.98	0.048	2.75 (1.01–7.48)
Hypoproteinemia					
0	–	–	–	–	1.00 (Reference)
1	1.65	0.54	3.08	0.002	5.23 (1.83–14.97)

CI = confidence interval, OR = odds ratio.

### 3.5. Plotting of nomograms

**Figure 1.**
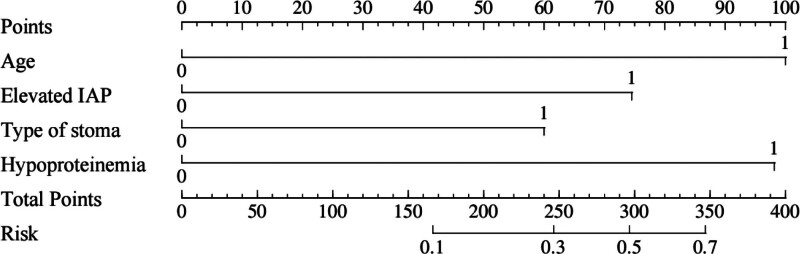
Nomogram prediction of the risk of stoma prolapse in colorectal cancer stoma patients in the six-month postoperative time period.

**Figure 2.**
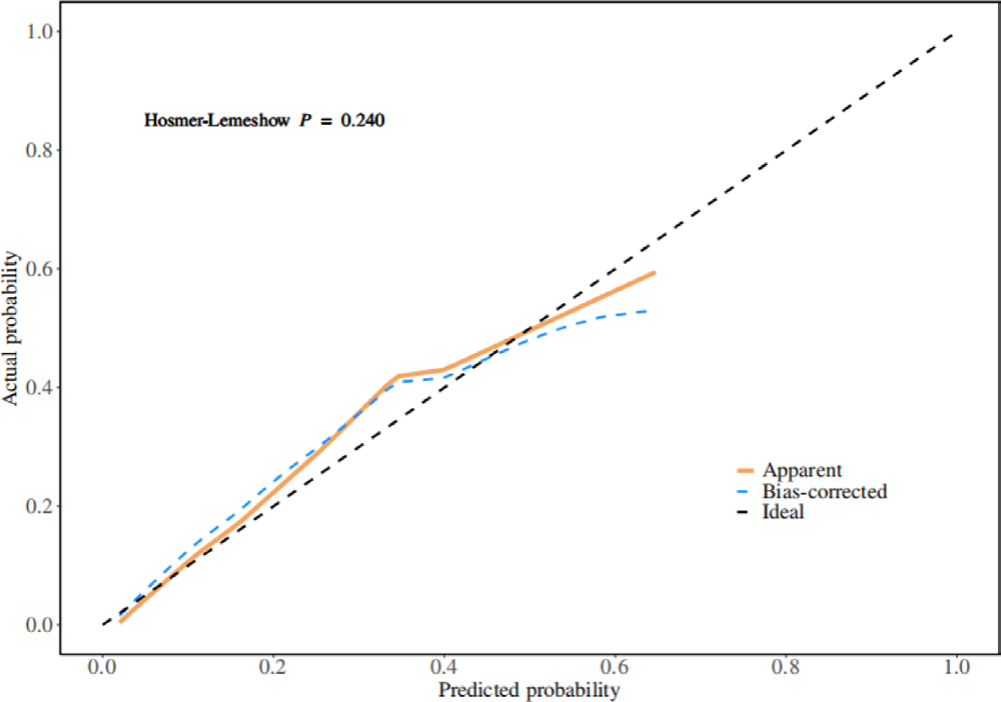
Internal validation of nomograms: calibration curves.

**Figure 3.**
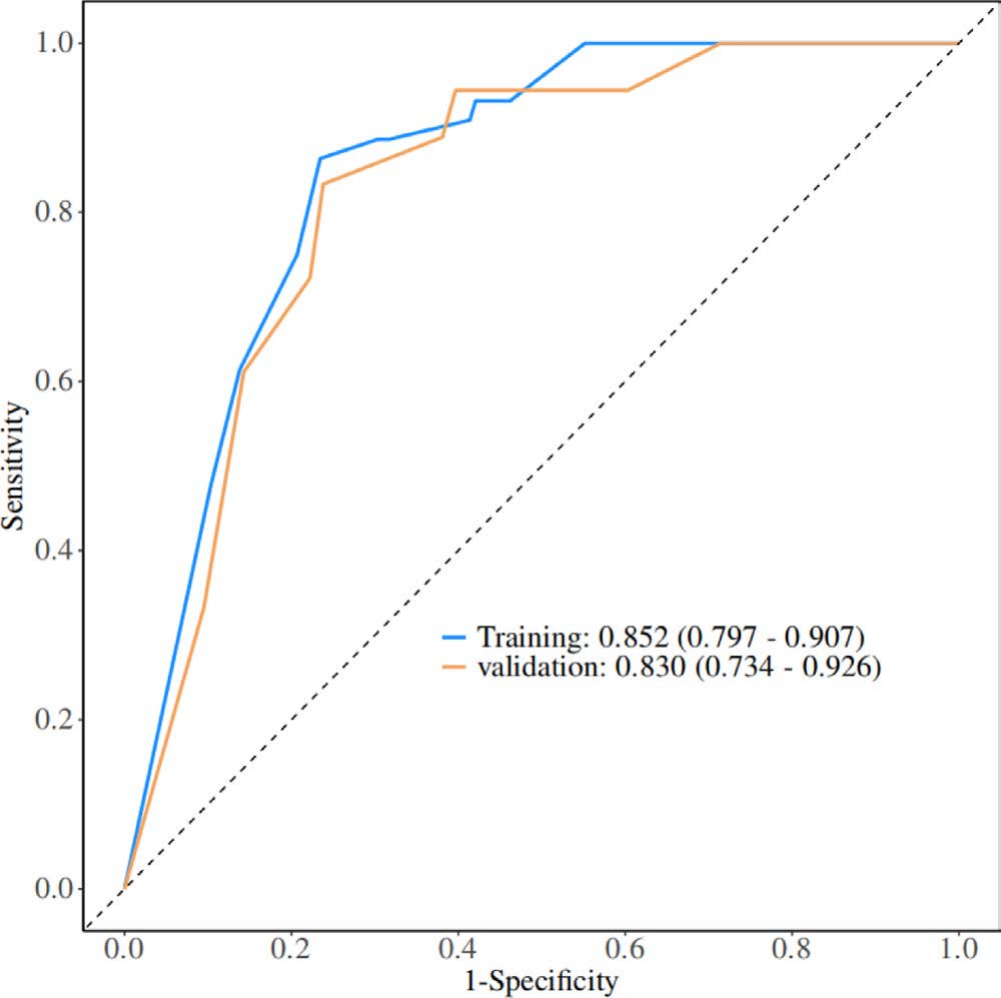
Validation of nomograms: ROC curves. ROC = receiver operating characteristic.

**Figure 4.**
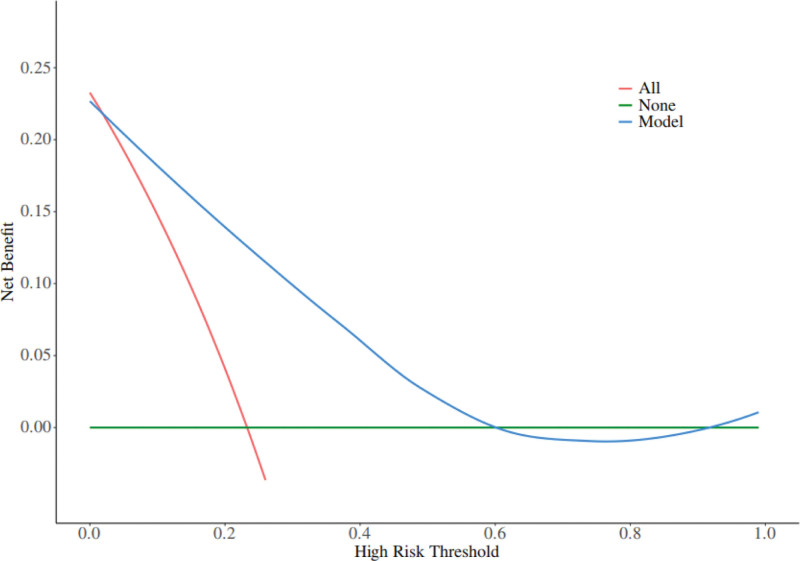
Decision curves in nomogram models.

A nomogram of the risk of stoma prolapse in patients with colorectal cancer stoma in the 6-month postoperative period was constructed based on 4 independent predictors tested by multifactorial logistic regression analysis, see Figure 1 for details. A Nomo score was assigned to each independent risk factor, which was summed to give a total score based on the clinical characteristics of that patient, positioned on the Total points axis. The value on the Risk axis corresponding vertically downwards is the probability of stoma prolapse in that patient. The score for each independent predictor corresponds to the score for each independent predictor. The total score for each subject was the sum of each independent predictor score. The probability of developing stoma prolapse was determined by the total score on the axis of risk of developing stoma prolapse in patients with colorectal cancer stoma during the 6-month postoperative period. The model was subsequently validated internally, and the internal validation was carried out by repeating the sampling of the nomograms 1000 times using the Bootstrap method in the R software. The calibration curve is close to the ideal curve, indicating that the nomogram predicts the incidence of stoma prolapse in colorectal cancer stoma patients during the 6-month postoperative period with a high degree of agreement with the actual incidence, reflecting a good predictive performance, see Figure 2 for details. The ROC curve for this nomogram training set has an AUC of 0.852 (95% CI = 0.797–0.907); Validation set ROC curve with an AUC of 0.830 (95% CI = 0.734–0.926), see Figure 3 for details. It is shown that the nomogram has a good discriminatory effect on patients with colorectal cancer stoma who are at high risk of developing stoma prolapse in the 6-month postoperative time frame. The decision curve (DCA) of the nomogram shows that the model provides more net benefit than the “all intervene” or ‘none intervene’ strategy when the threshold probability of an individual is >0.05, a finding that suggests that the column This conclusion suggests that the line plot model has good clinical value in predicting stoma prolapse in colorectal cancer stoma patients within 6 months after surgery, see Figure 4 for details.

## 4. Discussion

In the field of colorectal cancer treatment, although stoma surgery has brought a chance of survival for some patients, the quality of life and the effectiveness of rehabilitation have been seriously compromised by the thorny complication of postoperative stoma prolapse. In recent years, academic attention to colorectal cancer stoma prolapse has been increasing, but there are still many gaps in related research that need to be filled.^[1–3]^ Existing studies on the pathogenesis of colorectal cancer have mostly stayed at the level of the association of basic factors and lacked in-depth analysis of the specific pathophysiological processes of colorectal cancer. In terms of clinical prediction, the construction of models is fragmented and ineffective, which is difficult to meet the demand for accurate diagnosis and treatment. This study focuses on the clinical characteristics and constructs and validates the stoma prolapse prediction model, aiming to break through the limitations of traditional research, provide new ideas and powerful tools for clinical practice, and open up a new chapter in the accurate prevention and treatment of colorectal cancer stoma prolapse. In the following section, the value and significance of this study will be discussed in depth, focusing on the factors of model construction, performance, comparison with existing results, clinical application prospects and limitations of the study.

### 4.1. An in-depth analysis of independent risk factors for colorectal cancer stoma prolapse

The present study confirms that increasing age is a nonnegligible independent risk factor for colorectal cancer stoma prolapse. At the molecular biological level, the process of cellular senescence accelerates with age. The decline of intracellular mitochondrial function and the increased generation of reactive oxygen species cause damage to biological macromolecules such as cellular DNA, proteins, and lipids, affecting normal cellular metabolism and function. Fibroblasts, which are the main cells for collagen synthesis in peri-stoma supportive tissues such as skin and fascia, also decline in function with age.^[17]^ Relevant studies have shown that in aging fibroblasts, the level of collagen gene transcription decreases, and collagen type I and III synthesis decreases significantly, leading to a decrease in the content of tissue elastic fibers and disturbed fiber arrangement. This results in decreased elasticity of the tissues around the stoma, weakening the support of the intestinal tube and making it difficult to resist external forces such as intra-abdominal pressure, thus increasing the risk of stoma prolapse.^[18,19]^ In addition, chronically elevated intra-abdominal pressure plays a key role in the pathogenesis of stoma prolapse. According to the principle of hydrodynamics, when intra-abdominal pressure increases, an uneven pressure field is formed in the abdominal cavity, and the pressure on the peri-stoma area increases significantly. From the point of view of tissue mechanical properties, persistent high intra-abdominal pressure acts on the peri-stoma intestinal tubes and supporting tissues, which can cause mechanical damage.^[20]^ The plasma membrane layer and muscular layer of the intestinal tube are stimulated by long-term pressure, smooth muscle cells atrophy and collagen fibers break, resulting in thinning of the intestinal wall and reduced toughness. At the same time, uneven pressure distribution will cause the mechanical balance of the supporting structures such as fascia and muscles around the stoma to be broken, such as pelvic floor muscle relaxation, and the fixation of the intestinal tube is weakened. This mechanical damage accumulates and eventually causes the intestinal tube to prolapse from the stoma, triggering stoma prolapse.^[21]^ Secondly, studies have confirmed that the risk of prolapse varies between stoma types due to anatomical and functional differences. The end colostomy is a section of bowel that is cut off and the proximal bowel is pulled out of the abdominal wall to form a stoma, and the distal bowel is closed or sutured shut in the abdominal cavity.^[13]^ With this type of stoma, the intestinal tube has relatively high mobility and lacks effective wrapping and fixation by the surrounding tissues. Once the intra-abdominal pressure rises or the supporting tissues are weak, the intestinal tube is prone to dislocation and prolapse. In contrast, the loop stoma is to pull out a section of the intestinal tube through the abdominal wall, and cut the anterior and posterior walls of the intestinal tube outside the abdominal wall to form 2 openings, which resemble the shape of tabs.^[13]^ Compared with the end colostomy, the loop stoma is surrounded by more intestinal tubes and mesenteric tissues, which have a certain supporting and fixing effect on the intestinal tubes, and the risk of prolapse is relatively low. However, due to the more openings of the intestinal tubes, the structure and function of the surrounding tissues will be affected in the event of infection and inflammation due to improper care, which will indirectly increase the risk of prolapse.^[12,22]^

In addition to this, it has been shown that hypo-proteinaemia is an independent risk factor for stoma prolapse in patients with colorectal cancer stoma in the 6 months postoperative period, and that hypo-proteinaemia affects the state of the peri-stoma tissues through a number of biochemical pathways, which in turn increases the risk of stoma prolapse. Plasma colloid osmolality is maintained primarily by plasma proteins, particularly albumin. When hypoproteinemia occurs, plasma colloid osmolality decreases and intravascular water is transferred to the tissue interstitium, resulting in peri-stoma tissue edema. Tissue edema not only interferes with cellular nutrient exchange and metabolite elimination, resulting in impaired cellular metabolism, but also weakens tissue strength and elasticity. At the cellular level, the low protein state inhibits intracellular energy metabolic pathways such as the tricarboxylic acid cycle, affecting cell proliferation, differentiation and repair functions. For fibroblasts and endothelial cells around the stoma, their ability to synthesize extracellular matrix components such as collagen and fibronectin is reduced, resulting in impaired tissue repair. When the stoma is subjected to external force or fluctuation of intra-abdominal pressure, the tissues lacking good repair and support are more prone to prolapse.^[23]^

Not only that, stoma prolapse in colorectal cancer stoma patients in the 6 months postoperative period is also associated with many influencing factors. When patients have comorbid underlying diseases such as diabetes and cardiovascular disease, it can have a complex effect on stoma prolapse.^[12]^ Long-term hyperglycemia in diabetic patients can trigger vasculopathy, leading to endothelial cell damage in small and medium-sized blood vessels, thickening of basement membrane, narrowing of vascular lumen, and affecting the local blood transport of stoma. The impaired blood flow leads to tissue ischemia and hypoxia, insufficient supply of nutrients, and abnormal cellular metabolism, thus weakening the healing ability of the tissues and the ability to resist infection. At the same time, diabetes also causes peripheral neuropathy, resulting in peri-stoma nerve sensory loss, patients’ perception of local stoma discomfort is reduced, and potential problems cannot be detected and treated in a timely manner, increasing the risk of stoma prolapse. Cardiovascular diseases such as coronary artery disease and heart failure are often associated with cardiac insufficiency in patients, and obstruction of venous return can indirectly lead to increased intra-abdominal pressure, which can further exacerbate the pressure load on the tissues around the stoma. In addition, some of the medications used in the treatment of cardiovascular disease (e.g., vasodilators, etc) may affect vascular tone and tissue perfusion, which also has a potential impact on stoma recovery. In addition, differences in the surgical approach (open vs laparoscopic surgery) can lead to differences in local hematology and innervation of the stoma. Open abdominal surgery is highly traumatic and the extensive intraoperative separation of the abdominal wall and intra-abdominal tissues may damage the blood vessels and nerves surrounding the stoma. Vascular damage affects the local blood supply to the stoma and delays tissue healing, while nerve damage may lead to neuromodulation dysfunction of the muscles, fascia and other tissues around the stoma, making them less adaptable to changes in intra-abdominal pressure. Although laparoscopic surgery has the advantages of less trauma and faster recovery, the establishment of the pneumoperitoneum and the operation process may have a certain impact on the intra-abdominal pressure and haemodynamics, and when the stoma is operated laparoscopically, the operator’s operating skills are required to be higher, and if it is not operated correctly, it may affect the position and angle of the stoma as well as the fixation of the surrounding tissues, thus increasing the risk of stoma prolapse. In addition, different surgical methods also have an impact on the degree of postoperative pain and the recovery time of gastrointestinal function, which indirectly affect the patient’s activities and recovery process, and thus have a role in stoma prolapse.^[12]^ The nomogram model constructed in this study based on 4 independent predictors identified by multifactorial logistic regression analysis demonstrated unique advantages in predicting the risk of stoma prolapse in colorectal cancer stoma patients within 6 months after surgery. In terms of sensitivity, it reflects the ability of the model to correctly identify patients with stoma prolapse. The area under the curve (AUC) of the working characteristics of the subjects combined reflects the overall predictive accuracy of the model. In this study, the training set AUC was 0.852 (95% CI = 0.797–0.907) and the validation set AUC was 0.830 (95% CI = 0.734–0.926), both of which are at high levels. The closer the AUC is to 1, the better the model discriminates. When AUC = 0.5, the model predictive ability is no different from random guessing. The high AUC value of this model indicates that it has good differentiation between high-risk and low-risk groups of stoma prolapse in colorectal cancer stoma patients in the first 6 months after surgery, and has high practical value in clinical screening. For the high-risk group, the model can effectively warn the risk of stoma prolapse by accurately assigning Nomo scores to each independent risk factor. For example, a higher total score calculated by the model corresponds to a significantly higher probability of stoma prolapse on the Risk axis when the patient is older, has a long-standing condition of elevated intra-abdominal pressure (e.g., chronic cough, constipation that is not effectively controlled), and belongs to a specific high-risk type of stoma (e.g., telangiectomy) that is associated with hypo-proteinaemia. Based on this early warning, clinicians can prevent stoma prolapse from occurring by implementing intensive management for high-risk patients, such as closely monitoring stoma conditions, instructing patients to strictly control factors related to intra-abdominal pressure, and optimizing nutritional support protocols to correct hypo-proteinaemia. For the low-risk population, the model accurately excludes stoma prolapse high-risk status through a lower total score. Such patients are likely to be younger, have stable intra-abdominal pressure, relatively safe stoma type and good nutritional status. The negative predictive results of the model may enable clinicians to rationally arrange the follow-up schedule, avoid excessive medical interventions, save medical resources, and reduce the psychological burden of patients at the same time.

### 4.2. Preventive measures that can be taken against high-risk factors

Older people are more prone to stoma prolapse after surgery due to abdominal wall muscle atrophy, loss of collagen in the abdomen, and decreased healing ability. Albumin supplementation can be administered to elderly patients before and after surgery. This not only improves their nutritional status, promotes postoperative recovery, and accelerates rehabilitation, but also enhances their immunity, thereby reducing the risk of postoperative stoma prolapse due to local infection. In addition, some chronic comorbidities in older adults (such as constipation and chronic cough) need to be noted. When older adults defecate or cough, intra-abdominal pressure inevitably increases, which can exacerbate the risk of stoma prolapse. For patients with elevated intra-abdominal pressure, the most important thing is to treat the underlying conditions. For example, for patients with chronic constipation, polyethylene glycol 4000 can be administered for treatment. For patients with chronic cough, bronchodilators can be administered 1 month prior to surgery. Once the underlying conditions causing elevated intra-abdominal pressure have been appropriately managed, surgery can be performed to effectively avoid the impact of elevated intra-abdominal pressure on stoma prolapse. If the patient still has elevated intra-abdominal pressure after surgery, in addition to continuing treatment for the comorbidities causing the elevated intra-abdominal pressure, posture training can also be used. The head of the patient’s bed can be raised, and the patient can be kept in a semi-recumbent position (30–45°) after surgery to reduce the pressure on the diaphragm and abdominal cavity. If the patient still has uncontrollable coughing, cough control methods can be used. First, instruct the patient to take a deep breath, then hold their breath for 2 seconds, followed by 2 short, forceful coughs. This method maximizes the reduction of intra-abdominal pressure caused by coughing. The type of stoma is also associated with stoma prolapse following colorectal cancer surgery. For patients with colorectal cancer who require stoma surgery, a loop stoma should be performed whenever possible. If the patient has an end stoma, intestinal fixation surgery may be performed as necessary to reduce the risk of stoma prolapse. Additionally, during routine stoma care, nurses should handle the stoma gently to minimize irritation. If issues such as stoma retraction or collapse are detected, they should be addressed promptly. For patients with hypoalbuminaemia, human albumin preparations should be administered promptly. If the patient’s condition is severe, treatment measures such as blood transfusions may be taken.

### 4.3. Comparative analysis with previous research results

In the past, some prediction models for colorectal cancer stoma prolapse at home and abroad were mostly constructed by single-factor analysis or simple multifactor linear regression. Although such methods are easy to operate, it is difficult to comprehensively capture the complex interactions among factors. For example, models are constructed only on the basis of patient age and stoma type, ignoring the synergistic effects of key factors such as intra-abdominal pressure and nutritional status.^[24]^ In contrast, this study used multifactorial logistic regression analysis, which can effectively deal with the nonlinear relationship between multiple independent variables and the dichotomous dependent variable (the occurrence of stoma prolapse or not), and comprehensively consider the comprehensive impact of various factors on the risk of stoma prolapse. At the same time, this study uses data mining technology to analyze the clinical data in depth and filter out the most predictive value of the clinical features, so as to further optimize the construction of the model and make the model more in line with the actual clinical situation. In addition, some domestic studies have only focused on basic demographic characteristics of patients and surgery-related factors, such as only the age of patients and surgical methods were included in the model, and the local physiological status of the stoma and the nutritional and metabolic indicators of patients were not involved. In contrast, the present study innovatively included hypo-proteinaemia, a key indicator reflecting the nutritional and metabolic status of patients.^[25]^ Hypoproteinemia can lead to edema and reduced repair capacity of peri-stoma tissues, significantly increasing the risk of stoma prolapse, and the inclusion of this indicator fills the gap in previous models in the consideration of nutritional factors. In addition, this study refined the factors of elevated intra-abdominal pressure, not only focusing on whether the patient has chronic cough, constipation and other common symptoms that lead to increased intra-abdominal pressure, but also analyzing its duration, severity and other quantitative indicators, so that the model’s assessment of the factors of intra-abdominal pressure is more accurate and comprehensive.

Analysis of actual cases revealed that clinicians have limited accuracy in identifying high-risk patients with stoma prolapse in the absence of model assistance. The study showed that a large number of high-risk patients were missed due to empirical judgement that is highly subjective and easily overlooks factors such as the patient’s underlying connective tissue weakness and insidious chronic elevation of intra-abdominal pressure.^[26]^ Traditional empirical judgement is susceptible to the doctor’s personal knowledge base and richness of clinical experience. For example, young doctors may miss key risk factors in their judgement due to their relatively low exposure to stoma prolapse cases, resulting in missed diagnosis of high-risk patients. Based on objective data and rigorous statistical analysis, the model in this study is significantly better than traditional empirical judgement in identifying high-risk patients, which can effectively compensate for the judgement bias caused by doctors’ lack of experience. Traditional empirical judgement is highly subjective and susceptible to interfering factors. In clinical work, doctors may misjudge the risk of stoma prolapse due to factors such as the complexity of the patient’s condition and busy workload. For example, if the patient has multiple underlying diseases, the doctor may focus on the main diseases and ignore the risk factors related to stoma. In addition, different doctors attach different importance to risk factors, and there is a lack of uniformity in the judgement criteria, which leads to poor reliability of the judgement results. The present model provides a quantitative risk assessment system, assigning a clear Nomo score to each patient’s clinical characteristics and calculating the total score to derive the probability of stoma prolapse, which is free from subjective factors and makes the results more reliable and consistent. This model provides an objective and quantitative basis for clinical decision-making and greatly improves the standardization and precision of clinical diagnosis and treatment.

### 4.4. Model strengths, research limitations and prospects

The colorectal cancer stoma prolapse prediction model constructed in this study can be seamlessly embedded into the hospital’s existing electronic medical record system by developing a specialized clinical software plug-in (such as Donghua). When a doctor receives a patient with a colorectal cancer stoma, the system automatically extracts key clinical data such as the patient’s age, intra-abdominal pressure-related symptoms, stoma type, and proteaemia indicators, quickly calculates the total Nomo score, and visually demonstrates the patient’s probability of stoma prolapse. As the outpatient clinic is the first point of contact for patients with healthcare services, the model can play an initial screening role in this scenario. For newly diagnosed colorectal cancer patients who are to undergo stoma surgery, the model can be used by clinicians to quickly assess their risk of postoperative stoma prolapse. If the patient is judged to be at high risk, a specialized stoma care clinic can be arranged in advance for detailed guidance, including diet, exercise and precautions to prevent increased intra-abdominal pressure. At the same time, patients are booked for a closer follow-up programme to ensure early intervention and monitoring.

There are still some limitations in this study, and incomplete recording of some patients’ clinical information was more common during the data collection process. For example, some primary hospitals kept brief records of patients’ stoma care details, which may omit information such as the frequency of changing stoma bags and the condition of the skin around the stoma, thus affecting the accurate assessment of the local environment and potential risk factors of the stoma. In addition, The sample size of this study was small, and some risk factors associated with stoma prolapse after colorectal cancer surgery may not have been included. Therefore, this study was subject to a certain degree of selection bias. Meanwhile, measurement errors should not be ignored, such as intra-abdominal pressure measurement, different measurement tools and operation specifications may lead to data bias, which in turn affects the accurate judgement of the model on the intra-abdominal pressure factors and biases the model prediction results. This study did not set up a randomized controlled trial, which could not completely exclude the interference of confounding factors. For example, there are differences in the way patients are cared for by different medical teams and in postoperative rehabilitation instructions, and these factors may influence the occurrence of stoma prolapse, but it is difficult to control them accurately in the existing study design, which reduces the persuasiveness of the study results. Future studies may further expand the sample size to include colorectal cancer stoma patients from different geographical regions (e.g., different countries, regions with different levels of economic development) and different races. Patients in different regions have differences in living habits, use of medical resources, and disease spectrum, and different races may have different genetic backgrounds and physiological characteristics, all of which may affect the occurrence of stoma prolapse. By collecting a wide range of samples, the representativeness and generalisability of the model to different populations can be improved, and the value of the model for global application can be enhanced. In addition, patients with richer disease characteristics, such as patients with different tumor stages and different treatment options (differences in surgical approaches and radiotherapy regimens), were included. The risk of stoma prolapse varies with different conditions, such as local stoma status and overall body condition. Enriching the characteristics of the sample’s condition helps the model learn more comprehensive risk factors and improves prediction accuracy. Although this study took multiple measures to control for bias, such as using a double-blind review method to diagnose stoma fistula: 2 independent surgeons confirmed the diagnosis based on clinical photographs and medical records, and if there was a discrepancy, a third expert made the final decision; when collecting patient clinical data, we included as many potential risk factors as possible to reduce confounding bias that may be caused by unknown factors related to the outcome; The study population was clearly defined as patients who underwent radical colorectal cancer resection and stoma surgery between January 2021 and June 2024, excluding emergency surgeries, palliative surgeries, and cases involving simultaneous multi-organ resections to reduce heterogeneity. However, due to the inherent limitations of retrospective studies, this study may still be subject to a certain degree of bias. Future studies could conduct multicentre, large-sample prospective cohort studies to reduce the impact of bias.

## 5. Conclusions

The present study used multifactorial analysis and other statistically rigorous methods to analyze a large amount of clinical data, and clearly demonstrated that aging, abnormally high intra-abdominal pressure, specific stoma types, and hypoproteinemia, as independent risk factors, play a key role in the pathogenesis of stoma prolapse. Based on this finding, this study successfully constructed a clinical prediction model for stoma prolapse, which has demonstrated excellent efficacy in clinical practice, providing clinicians with an accurate prediction tool based on quantitative indicators. Through these precise interventions based on the model, it is expected that the pathological process of stoma prolapse can be blocked before the onset of the disease, and the incidence of stoma prolapse can be significantly reduced, thus improving the long-term prognosis and quality of life of patients with stoma.

## Author contributions

**Project administration:** Fei An.

**Writing – original draft:** Fei An, Lin Gui.

**Writing – review & editing:** Fei An, Yuting Li, Minjing Cheng.

## References

[1] Wu Y Li Y Wang X. Disability-adjusted life years for colorectal cancer in China, 2017-2030: a prevalence-based analysis focusing on the impact of screening coverage and the application of local weights. Chin Med J (Engl). 2025;14:e70592. 10.1097/CM9.0000000000003457 PMC12037099 40143427

[2] Hsieh CC Chen SY Lin CH Chen S-C Liao C-M. Disability-adjusted life years (DALYs) due to breast, cervical, colorectal and oral cancers in Taiwan Regions. Cancer Med. 2025;14:e70592. 39778066 10.1002/cam4.70592 PMC11705416

[3] Sung H Ferlay J Siegel RL. Global Cancer Statistics 2020: GLOBOCAN estimates of incidence and mortality worldwide for 36 cancers in 185 countries. CA Cancer J Clin. 2021;71:209–49. 33538338 10.3322/caac.21660

[4] Esmailzadeh A Fakhari MS Saedi N Shokouhi N Almasi-Hashiani A. A systematic review and meta-analysis on mortality rate following total pelvic exenteration in cancer patients. BMC Cancer. 2024;24:593. 38750417 10.1186/s12885-024-12377-5 PMC11095034

[5] Hilovsky D Hartsell J Young JD Liu X. Stable isotope tracing analysis in cancer research: advancements and challenges in identifying dysregulated cancer metabolism and treatment strategies. Metabolites. 2024;14:318. 38921453 10.3390/metabo14060318 PMC11205609

[6] Andre T Elez E Van Cutsem E; CheckMate 8HW Investigators. Nivolumab plus ipilimumab in microsatellite-instability-high metastatic colorectal cancer. N Engl J Med. 2024;391:2014–26. 39602630 10.1056/NEJMoa2402141

[7] Wang F Jin Y Wang M. Combined anti-PD-1, HDAC inhibitor and anti-VEGF for MSS/pMMR colorectal cancer: a randomized phase 2 trial. Nat Med. 2024;30:1035–43. 38438735 10.1038/s41591-024-02813-1

[8] Battaglin F Ou FS Qu X. HER2 gene expression levels are predictive and prognostic in patients with metastatic colorectal cancer enrolled in CALGB/SWOG 80405. J Clin Oncol. 2024;42:1890–902. 38457761 10.1200/JCO.23.01507 PMC11240881

[9] Papadopoulos V Bangeas P Xanthopoulou K Paramythiotis D Michalopoulos A. Stoma prolapse handmade repair under local anesthesia with variation of altemeier method in severe patients: a case report and review of the literature. J Surg Case Rep. 2017;2017:rjx027. 28458834 10.1093/jscr/rjx027 PMC5400450

[10] Siddiqui MT Shaukat F Khan MR Zahid N Arbani S. Quality of life of colorectal cancer patients and its association with anxiety and depression: cross-sectional study at a tertiary care hospital in low middle income country. J Surg Res. 2024;301:336–44. 39018953 10.1016/j.jss.2024.06.015

[11] Ya-Juan Z Fang-Hui D Yi-Wei X Gui-Fen LV San-Lian H Li-Li M. Comparative study of the risk prediction model of early postoperative frailty in elderly enterostomy patients based on machine learning methods. Front Med (Lausanne). 2024;11:1404557. 39045416 10.3389/fmed.2024.1404557 PMC11264199

[12] Garoufalia Z Mavrantonis S Emile SH. Surgical treatment of stomal prolapse: a systematic review and meta-analysis of the literature. Colorectal Dis. 2023;25:1128–34. 36965087 10.1111/codi.16548

[13] Gerçel G Azizoglu M Karakas E Risteski T Escolino M De La Torre L. Comparing loop and divided colostomy for anorectal malformation: a systematic review and meta-analysis. J Pediatr Surg. 2025;60:161974. 39358082 10.1016/j.jpedsurg.2024.161974

[14] Whiteside TL. The tumor microenvironment and its role in promoting tumor growth. Oncogene. 2008;27:5904–12. 18836471 10.1038/onc.2008.271 PMC3689267

[15] Shi Q Xue C Zeng Y. Notch signaling pathway in cancer: from mechanistic insights to targeted therapies. Signal Transduct Target Ther. 2024;9:128. 38797752 10.1038/s41392-024-01828-x PMC11128457

[16] Ordu M Demir E Tofallis C. A comprehensive modelling framework to forecast the demand for all hospital services. Int J Health Plann Manage. 2019;34:e1257–71. 30901132 10.1002/hpm.2771

[17] Bhatti JS Kumar S Vijayan M Bhatti GK Reddy PH. Therapeutic strategies for mitochondrial dysfunction and oxidative stress in age-related metabolic disorders. Prog Mol Biol Transl Sci. 2017;146:13–46. 28253984 10.1016/bs.pmbts.2016.12.012

[18] Zeng Q Gong Y Zhu N Shi Y Zhang C Qin L. Lipids and lipid metabolism in cellular senescence: emerging targets for age-related diseases. Ageing Res Rev. 2024;97:102294. 38583577 10.1016/j.arr.2024.102294

[19] Furth JJ. The steady-state levels of type I collagen mRNA are reduced in senescent fibroblasts. J Gerontol. 1991;46:B122–124. 2030266 10.1093/geronj/46.3.b122

[20] Mulita F Lotfollahzadeh S. Intestinal stoma. StatPearls. StatPearls Publishing LLC; 2025. 33351447

[21] Sato Y Tsujinaka S Shibata C. Seprafilm facilitates stoma closure by reducing peristomal/intra-abdominal adhesions. Asian J Surg. 2024. 10.1016/j.asjsur.2024.08.139 39227301

[22] Matsuki H Oura S Kataoka N. Successful Re-ileostomy using skin flap formation techniques. Cureus. 2024;16:e74940. 39744268 10.7759/cureus.74940 PMC11688590

[23] Takashima Y Hino H Shiomi A. Risk factors for stoma prolapse after laparoscopic loop colostomy. Surg Endosc. 2024;38:2834–41. 38605169 10.1007/s00464-024-10802-1

[24] Ba MQ Zheng WL Zhang YL. Construction of a nomogram prediction model for early postoperative stoma complications of colorectal cancer. World J Gastrointest Surg. 2025;17:100547. 39872787 10.4240/wjgs.v17.i1.100547 PMC11757204

[25] Lv Q Yuan Y Xiang Z. Analysis of risk factors for the sigmoid stoma complications in patients after abdominoperineal resection surgery: an observational study. Medicine (Baltim). 2024;103:e38751. 10.1097/MD.0000000000038751 PMC11466088 38941381

[26] Munshi E Segelman J Matthiessen P; RectoLeak Study Group. Increased risk of postoperative complications after delayed stoma reversal: a multicenter retrospective cohort study on patients undergoing anterior resection for rectal cancer. Int J Colorectal Dis. 2025;40:36. 39939486 10.1007/s00384-025-04831-y PMC11821667

